# Influence of Temperature on Molecular Adsorption and Transport at Liposome Surfaces Studied by Molecular Dynamics Simulations and Second Harmonic Generation Spectroscopy

**DOI:** 10.1021/acs.jpcb.1c04263

**Published:** 2021-09-08

**Authors:** Prakash Hamal, Visal Subasinghege Don, Huy Nguyenhuu, Jeewan C. Ranasinghe, Julia A. Nauman, Robin L. McCarley, Revati Kumar, Louis H. Haber

**Affiliations:** Department of Chemistry, Louisiana State University, Baton Rouge, Louisiana 70803-1804, United States

## Abstract

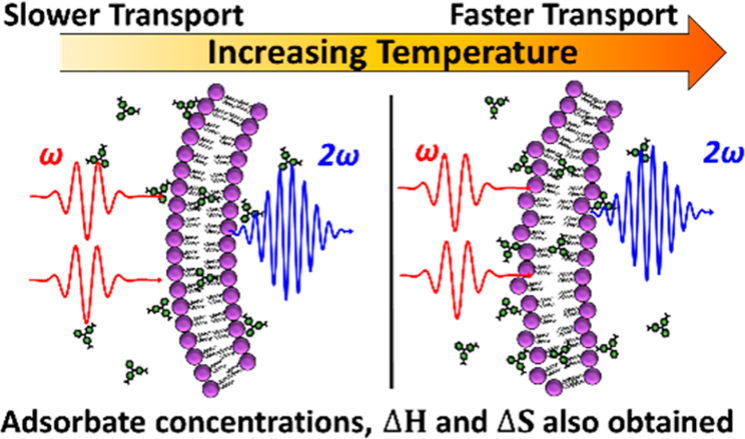

A fundamental understanding of the kinetics and thermodynamics of chemical interactions at the phospholipid bilayer interface is crucial for developing potential drug-delivery applications. Here we use molecular dynamics (MD) simulations and surface-sensitive second harmonic generation (SHG) spectroscopy to study the molecular adsorption and transport of a small organic cation, malachite green (MG), at the surface of 1,2-dioleoyl-*sn*-glycero-3-phospho-(1′-*rac*-glycerol) (DOPG) liposomes in water at different temperatures. The temperature-dependent adsorption isotherms, obtained by SHG measurements, provide information on adsorbate concentration, free energy of adsorption, and associated changes in enthalpy and entropy, showing that the adsorption process is exothermic, resulting in increased overall entropy. Additionally, the molecular transport kinetics are found to be more rapid under higher temperatures. Corresponding MD simulations are used to calculate the free energy profiles of the adsorption and the molecular orientation distributions of MG at different temperatures, showing excellent agreement with the experimental results.

## Introduction

Cellular membranes are constructed from a complex system of lipid species and membrane proteins for the regulation of molecular exchange with the surrounding environment.^1−3^ A comprehensive study of cellular membranes, including their organization and chemical interactions, is critically important in describing metabolic functions, signal transduction, and the translocation of molecules.^4−6^ Translocation can be characterized by molecular adsorption to the membrane surface and transport through the lipid bilayer.^7−9^ Liposomes are small vesicles composed of phospholipids and are considered to be models for studying more complex membranes in biological systems.^10−12^ Liposomes are also used in drug delivery^13^ and are related to lipid nanoparticles used to encapsulate mRNA in COVID-19 vaccines.^14^ Liposomes offer the potential to carefully examine the effect of temperature on the properties of molecular translocation across phospholipid membranes in aqueous solution. The thermodynamics and kinetics related to molecular adsorption and transport at liposome surfaces provide detailed information that is relevant for understanding chemical interactions between small molecules and biological membranes. Recent work has investigated the effect of temperature on molecular transport of small molecules through the liposome membrane.^15,16^ However, a complete characterization of molecular adsorption and transport in lipid bilayer systems, including the associated kinetics and thermodynamics, has not been studied in detail.

Second harmonic generation (SHG) spectroscopy has been widely used as an experimental method to probe the interfacial properties of colloidal nanoparticle systems.^11,17−24^ SHG is a nonlinear optical process where two photons with a frequency of ω add coherently to form a third photon with a frequency of 2ω. SHG is noninvasive, nondestructive, and surface-sensitive.^19,20^ The second harmonic response from isotropic, centrosymmetric bulk media is dipole forbidden, resulting in no coherent signal.^25,26^ However, an SHG signal is allowed at surfaces and interfaces where the symmetry is broken.^17,18,23,27−32^ SHG has been used extensively to study the molecular adsorption and transport of small cationic molecules, such as malachite green (MG)^22,33−36^ and malachite green isothiocyanate (MGITC),^12^ at liposome surfaces in aqueous solution. The adsorption of these dye molecules to the outer lipid bilayer produces an enhanced SHG signal,^33,37,38^ followed by a decrease in the SHG signal as the dye molecules transport through the membrane.^19,21,33,35,37−43^ Since the lipid bilayer thickness, which is about 5 nm, is much smaller than the SHG coherence length, dye molecules adsorbed at the inner and outer liposome interface produce cancellation in the SHG signal, providing a time-resolved method for measuring molecular transport.^19,44^ In our previous work, we investigated the molecular adsorption and transport properties of MG in liposomes with different lipids, buffers, and electrolyte conditions using time-dependent SHG.^11^ Added electrolytes can shield electrostatic interactions leading to decreased adsorption, altered adsorbate–adsorbate repulsion at the liposome surface, and increased ion-pairing causing longer transport times.^11,36^ We also studied the molecular interactions in different liposomes with the similar triphenylmethane dye molecule MGITC to examine the impact of chemical functional groups in these complicated translocation processes.^12^ A fundamental understanding of the factors affecting lipid-based delivery systems can lead to potential clinical applications where the release of drug molecules from a liposome can be influenced by the surface chemistry and changes in the local environment.

In this paper, we extend our investigations of chemical interactions at model biological membranes using time-dependent SHG spectroscopy combined with molecular dynamics (MD) simulations to study the molecular adsorption and transport of the small, drug-like cationic molecule malachite green with 1,2-dioleoyl-*sn*-glycero-3-phospho-(1′-*rac*-glycerol), DOPG, liposomes at various temperatures. Measuring the temperature-dependent adsorption isotherms using SHG provides for the determination of the free energies of adsorption and the adsorbate site concentrations, which further allows for the changes in enthalpy and entropy of the associated adsorption process to be obtained. Additionally, the kinetics of MG transport through the DOPG bilayer are measured, with the transport time decreasing as the temperature is increased, in agreement with previous studies.^16^ These thermodynamic and kinetic results provide additional and complementary information as compared to more conventional techniques^45−47^ due to the nonlinear optical surface sensitivity of SHG measurements. Corresponding temperature-dependent MD simulations are used to calculate the free energy profiles of bringing the MG molecule to the DOPG membrane, as well as the orientational distribution of MG at the bilayer surface, showing excellent agreement with the experimental results. By the combination of temperature-dependent and time-dependent SHG spectroscopy with MD simulations, the complicated chemical interactions occurring at the lipid bilayer interface in water are carefully studied for developing a greater understanding of biologically relevant molecular translocation at model cellular membranes.

## Methods

### Second Harmonic Generation Setup

The experimental setup for the SHG measurements has been described previously.^11,12,48^ A titanium:sapphire oscillator laser, with an output wavelength centered at 800 nm with a 75 fs pulse duration and 80 MHz repetition rate, is attenuated to an average power of 1.0 W and is focused to the sample contained in a 1 cm × 1 cm quartz cuvette to produce the SHG signal. The cuvette is wrapped with heating tape and monitored with a thermocouple to control the sample temperature while the time-dependent SHG intensity in the forward direction is measured using a high-sensitivity charge-coupled device (CCD) detector connected to a monochromator spectrograph. A magnetic stir bar is used for automated stirring, and a computer-controlled beam block is employed for measuring the background-subtracted SHG spectrum as a function of time for each liposome sample at each temperature and added MG concentration. MG is added rapidly to the liposome sample using a pipet in approximately 1 s for each measurement. A home-built data acquisition program collects 10 SHG spectra and 5 background spectra using 1 s acquisition times in repeating iterations to obtain the background-subtracted SHG time scans with statistical analysis.

### Synthesis and Characterization

The synthesis of large unilamellar vesicles (LUV) or liposomes of DOPG lipids has been previously reported^49,50^ and is discussed in more detail in the Supporting Information. DOPG was purchased from Avanti Polar Lipids, Inc. in powder form. Citric acid monohydrate (≥99.0%), potassium hydroxide purified pellets (≥85%), and malachite green chloride were purchased from Sigma-Aldrich. The molecular structure of the malachite green cation, C_23_H_25_N_2_^+^, is shown in Figure S4a of the Supporting Information. The colloidal liposomes are characterized using dynamic light scattering (DLS) and zeta-potential measurements using a Zetasizer Nano ZS from Malvern Instruments Inc., U.K. All measurements of the DOPG liposomes in this study are conducted in 5 mM citrate buffer with pH 4.0. The average diameter of the DOPG liposomes is measured to be 137 ± 42 nm with a polydispersity index of 0.07. Similarly, the corresponding zeta potential is determined to be −73.2 ± 1.1 mV.

### Molecular Dynamics Simulations Details

Molecular dynamics simulations are carried out with the all-atom general AMBER force field (GAFF)^51^ using the LAMMPS (version 05 Sep 2014)^52^ software. The molecular structures of the molecules are optimized, and the partial charges of the structures are calculated by the RESP fitting technique^53,54^ using the HF/6-31G* method in the Gaussian 09 suite of programs.^55^ The dye molecule–lipid system of MG with DOPG is simulated at two different temperatures of 303 and 313 K. The equilibration simulations are carried out under isothermal–isobaric (NPT) conditions followed by simulations in the canonical (NVT) ensembles. The final simulation box dimensions are approximately 94.5 Å × 50.0 Å × 115.0 Å for the system simulated at 303 K and 94.0 Å × 50.0 Å × 117.5 Å for the system simulated at 313 K. The membrane/water interface is perpendicular to the *z*-axis for each system. Additional details regarding the two systems and the simulation setup are discussed in the Supporting Information. Using the umbrella sampling method,^56^ the free energy profiles of bringing the MG molecule onto the DOPG membrane at the two different temperatures are determined. The displacement along the *z*-direction between the center of mass (COM) of the lipid bilayer and the COM of the dye molecule is used as the collective variable for the umbrella sampling. For both systems, 32 umbrella sampling windows are generated with an 18 ns simulation time per window and with a spacing of 1.5 Å along the *z*-axis. Using the weighted histogram analysis method (WHAM),^57,58^ the potential of mean force of the adsorption process is calculated using the last 14 ns of each umbrella sampling window. The statistical error of the potential of mean force is determined using the block averaging method with 3.5 ns of data for each block. The orientation of the dipole moment of the MG molecule with respect to the DOPG membrane as the dye approaches the membrane is analyzed and compared for the two temperatures. In addition, the number of water molecules within 3.5 Å in the *z*-direction of the average surface of the DOPG molecules, as defined using the outermost oxygen atoms of the DOPG molecules, is calculated for each umbrella sampling window for both temperatures to determine the number of water molecules displaced as the MG molecule penetrates the membrane. Additional details of these calculations and analysis for each temperature are summarized in the Supporting Information.

## Results and Discussion

**Figure 1:**
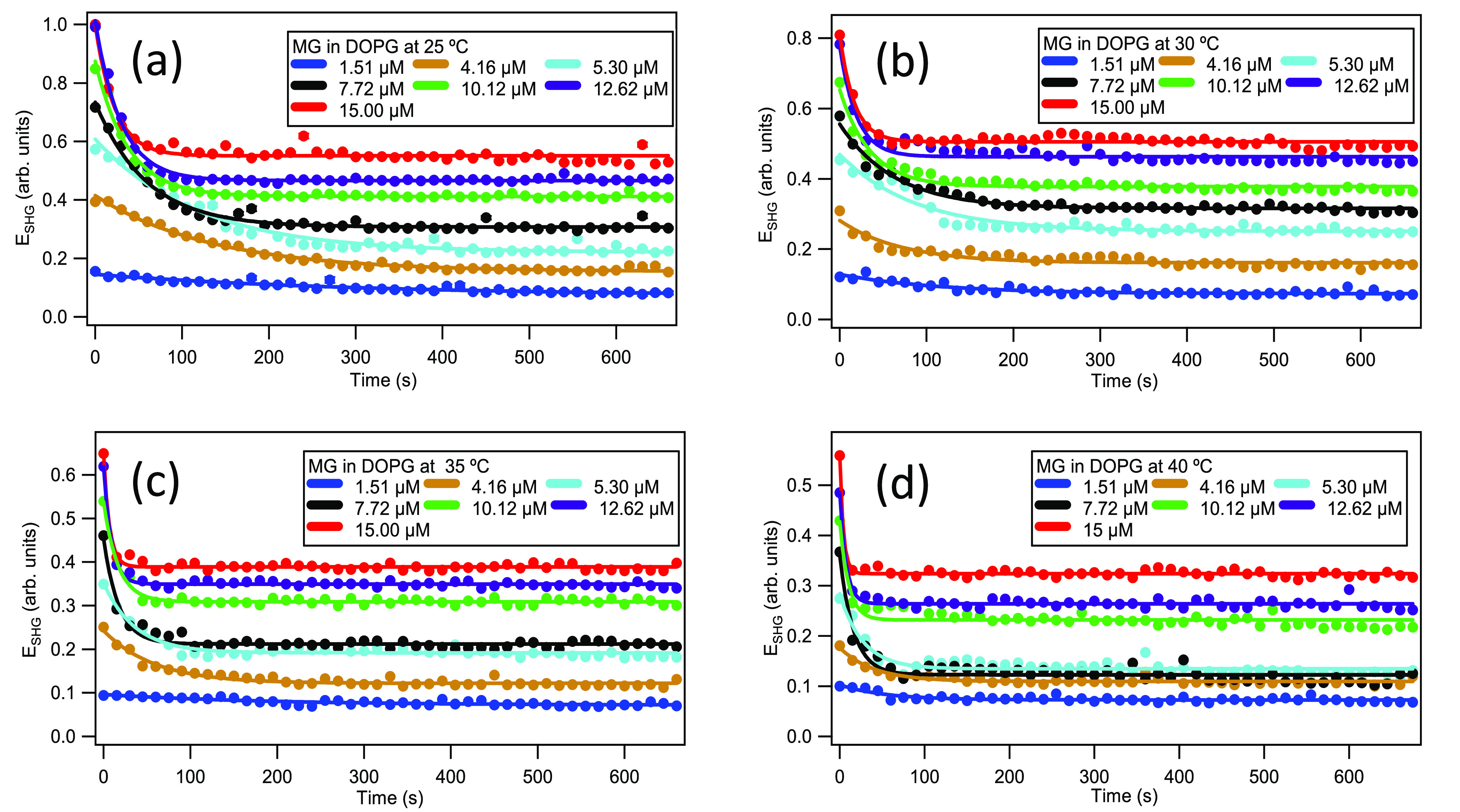
SHG time profiles upon addition of various concentrations of MG to 50 μM DOPG liposomes in 5 mM citrate buffer with pH 4.0 at (a) 25, (b) 30, (c) 35, and (d) 40 °C, respectively. Solid lines are best fits.

Time-dependent SHG measurements under varying sample temperature and MG concentration provide crucial information on the molecular transport through the lipid bilayer. The SHG signal *I*_SHG_ is observed to rise abruptly upon the addition of MG into the colloidal DOPG liposome sample due to MG adsorption to the outer surface of the bilayer, followed by a gradual, time-dependent exponential decrease in SHG signal caused by the MG transport through the liposome bilayer. These general observations are in agreement with previous studies.^11,12,33,35,59^ The transport kinetics of MG crossing the DOPG lipid bilayer are analyzed by fitting the time-dependent SHG electric field, where , using the exponential function, *E*_SHG_(*t*) = *a*_0_ + *a*_1_e^–*t*/τ^, to obtain the molecular transport time τ under different MG concentrations and bulk temperatures. Here, *t* is the experimental time after MG addition. These exponential fits are plotted as solid lines for each temperature and MG concentration, as shown in Figure 1. For a direct comparison, all SHG intensities are normalized with respect to the DOPG liposomes immediately upon addition of 15 μM MG at 25 °C. Representative SHG spectra and calculated *R*^2^ values for the exponential fits are included in the Supporting Information. The bulk temperatures used are all above the transition temperature *T*_m_ of −18 °C for DOPG, where the ordered gel phase changes to the more disordered liquid crystalline phase.^33^

**Figure 2:**
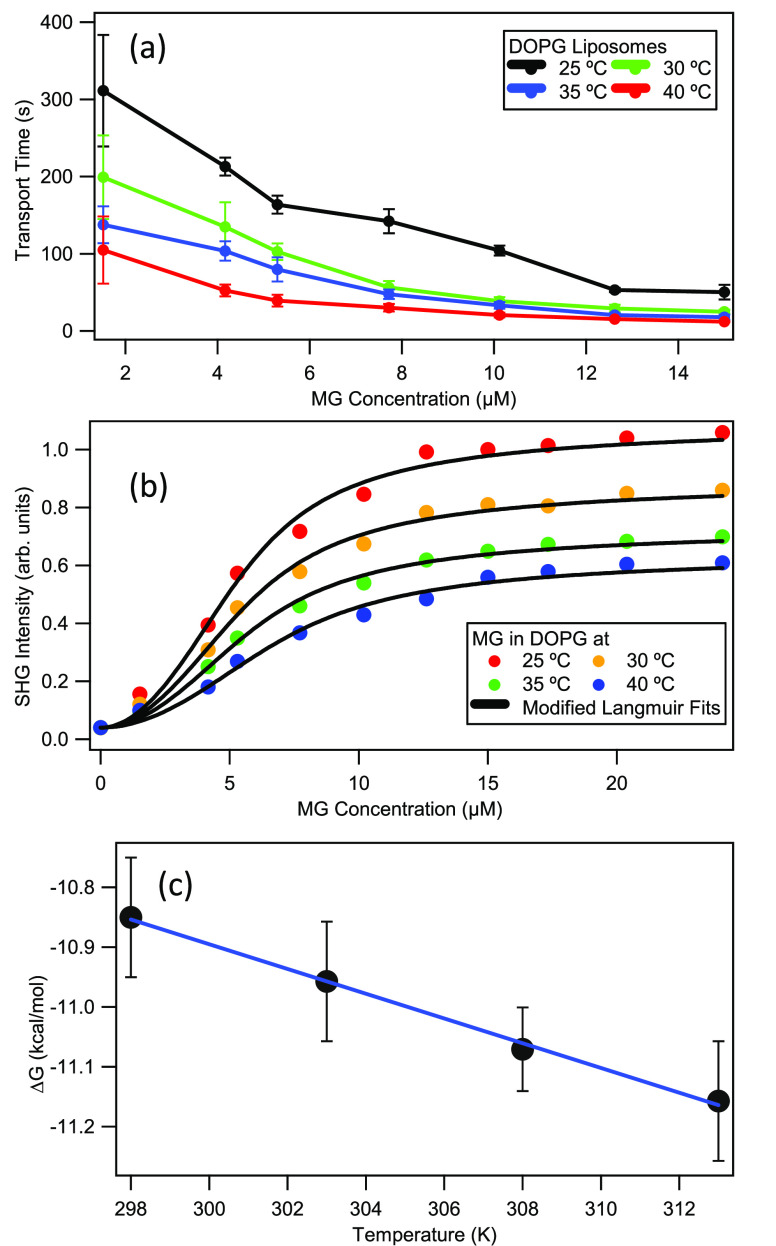
(a) Transport times as a function of MG concentration for DOPG liposomes at different temperatures. (b) SHG-determined adsorption isotherms for MG with DOPG liposomes in 5.0 mM citrate buffer at different temperatures. Solid lines are best fits. (c) Adsorption free energy for MG with DOPG liposomes as a function of temperature (black circles) with best linear fit (blue line).

The obtained transport times τ are plotted as a function of MG concentration for each temperature, as displayed in Figure 2a. The rate of molecular transport is significantly faster at higher temperatures. Applying heat to a lipid bilayer leads to increased hydrocarbon chain motion, less hydrogen bonding between adjacent acyl groups, and a larger volume of the overall nonpolar region.^60,61^ Similarly, the membrane fluidity is also increased at higher temperatures, which aids the rate of transport.^62,63^ These previous findings are consistent with our SHG results, where increased temperature leads to the observed decrease in the MG transport time. Our SHG results here are also in general agreement with previous SHG studies where increased temperature leads to faster transport times.^15,16^ Additionally, the obtained rate constants from the fits are shown to vary linearly as a function of MG concentration for each temperature,^11,35^ and these corresponding slopes are observed to vary linearly with temperature, as shown in the Supporting Information. The linear dependence of the rate constant with respect to temperature is analogous to the related process of temperature-dependent diffusion described by the Stokes–Einstein equation.^64^

Adsorption isotherm measurements are performed by measuring the SHG intensity as a function of MG concentration to obtain the adsorption site density and adsorption free energy for each sample temperature. For these isotherms, the SHG intensity is measured directly upon MG addition at *t* = 0 using a fresh liposome sample for each MG concentration and temperature. The experimentally obtained isotherms are fit using the modified Langmuir isotherm model to account for the reduction of the bulk concentration of the adsorbate molecules due to the large cumulative surface area of the colloidal liposome sample. The modified Langmuir model is given by^23,43^
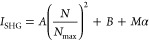
1and
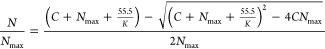
2where *N*_max_ is the maximum adsorption site concentration, *K* is the adsorption equilibrium constant, *A* is the SHG intensity at saturation, and *C* is the added dye concentration. Additionally, *N* is the concentration of dye molecules adsorbed, *B* is the baseline offset, *M* is the concentration of free dye molecules in solution, and α is the slope obtained from free dye molecules alone in solution as a function of *C*. Here, *N*_max_ and *K* refer to the adsorption to the outer liposome surface only, before molecular transport occurs. The fits of the modified Langmuir model to the experimental results are shown in Figure 2b, and the corresponding fit parameters are listed in Table 1. The adsorption equilibrium constant *K* is observed to decrease as the temperature is increased. This may be a consequence of increased counterion adsorption to the Stern layer under higher temperatures leading to decreased electrostatic attraction of MG to the bilayer surface. Additionally, the adsorption site densities are found to have an opposite behavior, where higher temperatures have increased maximum adsorption site concentration *N*_max_ values. This is attributed to the increased mobility of counterions and the increase in area of the lipid headgroups at higher temperatures.^62,63^ Increased counterion concentration at the bilayer surface at higher temperatures also shields adsorbate–adsorbate repulsion, contributing to the higher *N*_max_ values.^11^

**Table 1:** Fitting Parameters and Free Energies Obtained from the Modified Langmuir Model

	25 °C	30 °C	35 °C	40 °C
*K* (×10^7^)	9.1 ± 0.1	8.1 ± 0.2	7.2 ± 0.1	6.2 ± 0.3
*N*_max_ (μM)	5.4 ± 0.2	5.7 ± 0.4	6.0 ± 0.1	6.9 ± 0.3
*A*	1.0 ± 0.01	0.85 ± 0.01	0.69 ± 0.01	0.60 ± 0.01
–Δ*G* (kcal/mol)	10.8 ± 0.1	10.9 ± 0.1	11.1 ± 0.1	11.2 ± 0.1

The free energy of adsorption, obtained from Δ*G* = −*RT* ln *K*, is plotted as a function of temperature as shown in Figure 2c. The results are fit to a line with Δ*G* = Δ*H* – *T*Δ*S* to provide the thermodynamic properties of the molecular adsorption to the liposome surface, where Δ*H* is the change in adsorption enthalpy, Δ*S* is the change in adsorption entropy, and *T* is the temperature. The calculated Δ*H* from the *y*-intercept is −4.685 ± 0.326 kcal/mol, indicating that the net change in adsorption enthalpy represents an exothermic process. The calculated Δ*S* from the linear slope is 0.021 ± 0.002 kcal/K·mol. This change in entropy is a full accounting of the adsorption process, including the change in entropy of the adsorbate molecules as well as the overall liposome surface. The molecular adsorption by itself should have a negative change in entropy as the dye molecules are more ordered when adsorbed to the liposome surface. However, the adsorbate molecules replace water molecules and counterions that were originally at the liposome surface. Since each MG adsorbate molecule replaces numerous water molecules and counterions due to their relative sizes, an overall increase of entropy occurs upon adsorption when given a full account of all constituents. Our MD simulations indicate that approximately 70 water molecules are replaced by each MG molecule upon adsorption, as discussed in more detail in the Supporting Information. Here, because Δ*H* is negative and Δ*S* is positive, the process of MG adsorption to the DOPG liposome surface is expected to be spontaneous at all aqueous temperatures. These SHG results on adsorption thermodynamics and transport kinetics go beyond more conventional techniques, such as isothermal titration calorimetry^45,46,65^ and specialized fluorescence spectroscopy,^47^ because the adsorption and transport processes are directly observed and differentiated due to the nonlinear optical surface sensitivity. A comparative study of MG adsorption to colloidal polystyrene sulfate microspheres in water using temperature-dependent SHG measurements is included in the Supporting Information, demonstrating the general applicability of this technique for determining the thermodynamics of adsorption for a wide variety of colloidal systems.

**Figure 3:**
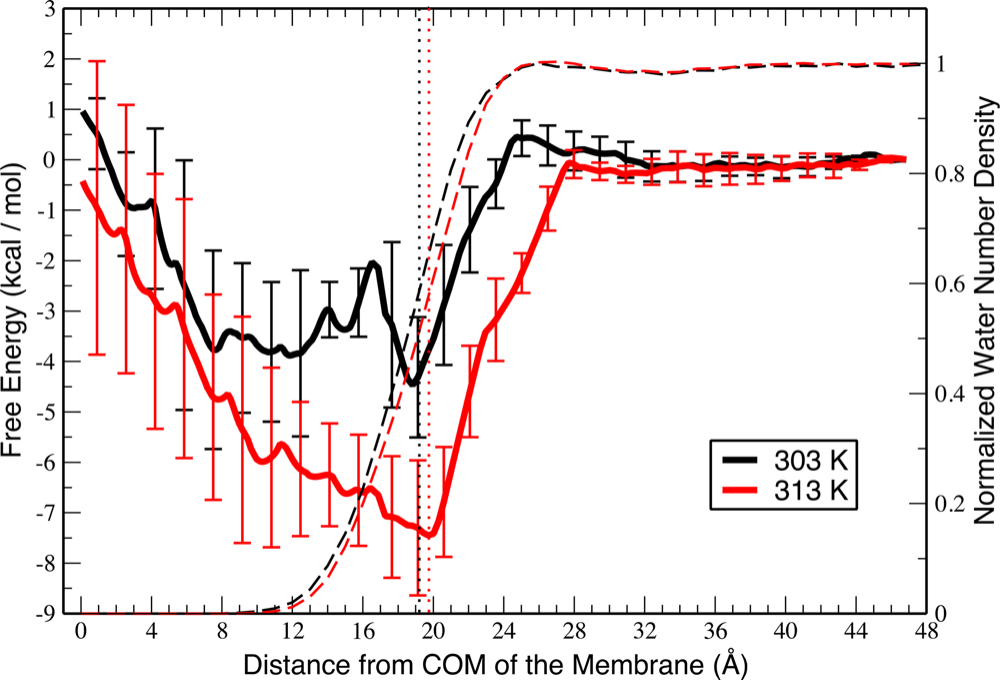
Potential of mean force curves for the adsorption process of the MG molecule to the DOPG lipid bilayer at temperatures of 303 and 313 K as a function of distance along the *z*-axis (which is perpendicular to the membrane–water interface) between the center of mass (COM) of the membrane and the COM of the dye. The vertical dotted lines represent the average interface for 303 K (black) and 313 K (red). The dashed lines represent the normalized water number density calculated when the dye is far away from the interface for 303 K (black) and 313 K (red).

Molecular dynamics simulations are used to obtain additional information about the interactions of MG molecules at the DOPG bilayer surface at two different temperatures. The free energy profiles for the adsorption of MG as a function of distance in the *z*-direction between the COM of the MG molecule and the COM of the DOPG membrane at the two different temperatures are displayed in Figure 3. According to the free energy profiles, at 313 K this adsorption process is essentially barrierless, whereas at 303 K there is a small energy barrier of ∼0.5 kcal/mol (∼2.1 kJ/mol) for the adsorption process. Although MG is thermodynamically more favored to be adsorbed on the membrane under both temperatures, the results in Figure 3 demonstrate that the stability of the MG molecule inside the membrane is greater at higher temperatures. In addition, during the initial equilibrium MD canonical simulations before performing the umbrella sampling simulation, the MG molecule is seen to rapidly adsorb and penetrate the DOPG membrane at the higher temperature, whereas the adsorption process takes much longer and displays less penetration at the lower temperature simulation as discussed in the Supporting Information. These results are consistent with the SHG experimental observations, where MG is transported faster through the bilayer and the corresponding Δ*G* value is more negative at higher temperatures. Future work is needed to investigate additional factors, such as the mechanism responsible for concentration-dependent transport times^11,35^ and the possibility of transient pore formation in the lipid bilayer.^66,67^

These SHG and MD studies also give important insight regarding the adsorbate ordering and orientational distributions at the colloidal liposome surface in water. It is well established that the SHG signal from colloidal nanoparticles depends on the orientation angle of adsorbates, as well as the scattering angle and polarization configuration.^29,68−70^ For our study, the SHG intensity at saturation *A* depends on both *N*_max_ and the orientational distribution of adsorbed MG at the liposome surface.^11,35,71^ Our experimental results show that *A* decreases as the temperature is increased, even as *N*_max_ is observed to increase. This suggests that the orientational distribution should also change as a function of temperature, leading to the lower measured SHG signals. Our MD simulation results are in excellent agreement with these observations, where the orientational distribution of the dipole moment of an adsorbed MG molecule broadens significantly at the higher temperature, leading to decreased ordering and lower SHG signals, as discussed in greater detail in the Supporting Information. Previous studies on MG in DOPG liposomes showed a more constant time-zero SHG signal under varying temperatures.^16^ However, these measurements were conducted at a 90° collection angle, while our measurements are performed in the forward direction, where different relative signal strengths from changes in *N*_max_ and orientational distribution could explain the differences in these experimental observations. Overall, the SHG studies of MG adsorption and transport in DOPG liposomes at various temperatures provide detailed information on fundamental chemical interactions at a lipid bilayer in water that is complementary and consistent with the corresponding MD simulations.

## Conclusion

The adsorption and transport of the small, cationic drug-like molecule malachite green at the DOPG liposome surface in water are investigated using molecular dynamics simulations and time-dependent second harmonic generation spectroscopy. The MD simulations results are in excellent agreement with the experimental results, demonstrating that the rate of transport is faster at higher temperatures. Additionally, the SHG adsorption isotherm measurements indicate that the adsorbate concentration increases at the liposome surface while the free energy of adsorption becomes more negative as the temperature is increased. By plotting the free energy as a function of temperature, the changes in enthalpy and entropy are obtained, showing that the adsorption process is exothermic with increasing entropy when taking a full account of all substituents. The MD simulations also determine the temperature-dependent free energy curves of adsorption, the orientational distributions of the adsorbate at the surface, and the number of water molecules displaced upon adsorption, which all provide an important context for interpreting the SHG results. In summary, this study shows that temperature is a critical and sensitive factor in quantifying chemical interactions with lipid bilayers, providing fundamental insight that can help in developing potential membrane-based drug-delivery applications.

## Supporting Information Available

Additional molecular dynamics simulations results, liposome characterization measurements, and details on SHG studies of molecular interactions with liposomes and polystyrene microspheres, including fit parameters and error analysis (PDF)
