# Supplementary material for: Influence of Temperature on Molecular Adsorption and Transport at Liposome Surfaces Studied by Molecular Dynamics Simulations and Second Harmonic Generation Spectroscopy
Source: J Phys Chem B. 2021 Sep 8;125(37):10506–13. doi: 10.1021/acs.jpcb.1c04263 (PMC8474114; doi:10.1021/acs.jpcb.1c04263)
Supplement: Supplementary file 1 — jp1c04263_si_001.pdf [file jp1c04263_si_001.pdf]

Supporting Information For

Influence of Temperature on Molecular Adsorption and Transport at Liposome Surfaces Studied by Molecular Dynamics Simulations and Second Harmonic Generation Spectroscopy

Prakash Hamal, Visal Subasinghege Don, Huy Nguyenhuu, Jeewan C. Ranasinghe, Julia A. Nauman, Robin L. McCarley, Revati Kumar, and Louis H. Haber*

Department of Chemistry, Louisiana State University, Baton Rouge, Louisiana 70803-1804, United States

*Corresponding author's email: lhaber@lsu.edu

Lipid Synthesis, Concentration Determination, and Liposome Size Distributions

The 1,2-dioleoyl-sn-glycero-3phospho-(1'rac-glycerol) (DOPG) liposome formation protocol has been reported previously.[1-3] For determination of the lipid concentration, perchloric acid was used to convert phospholipids to inorganic phosphates. The addition of ammonium molybdate in an acidic condition leads to the formation of phosphor-molybdic acid, which was then reduced by Fiske-Subbarow agent, producing a blue solution for absorbance measurements at 800 nm (Bartlett assay). Absorption measurements were done using a UV−vis spectrometer from PerkinElmer, Boston, MA, U.S.A. The calibration curve for the Bartlett assay, as shown in Figure S1, is fit with a linear equation with a slope of 0.00300 ± 0.00004, a y-intercept of −0.002 ± 0.003, and an R^2 value of 0.999 for the fit. The dynamic light scattering (DLS) size distribution is shown in Figure S2 with a size of 137 ± 42 nm and a polydispersity index of 0.07 for the DOPG liposomes in 5 mM citrate buffer with pH 4.0. The molecular structure of DOPG is shown in Figure S3.

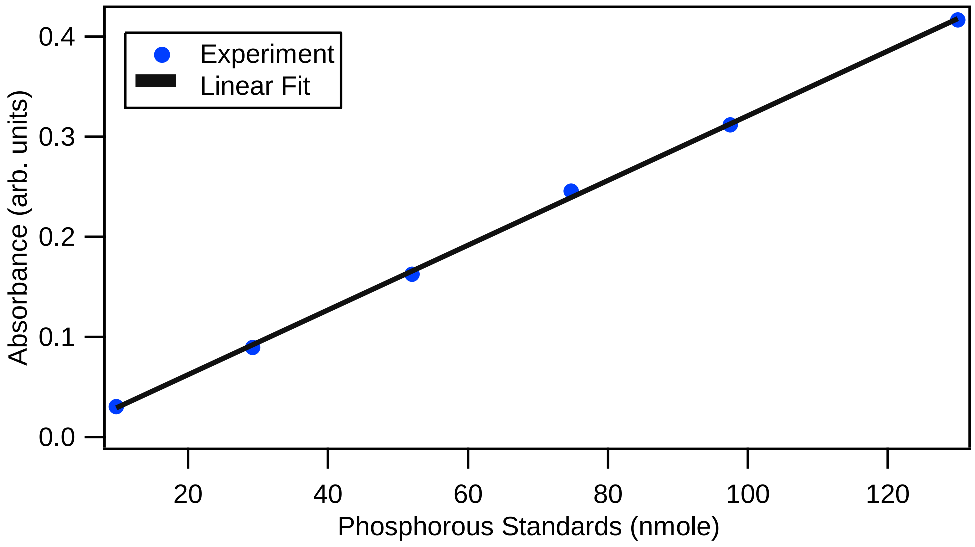

Figure S1: Bartlett assay calibration for determining the lipid concentration.

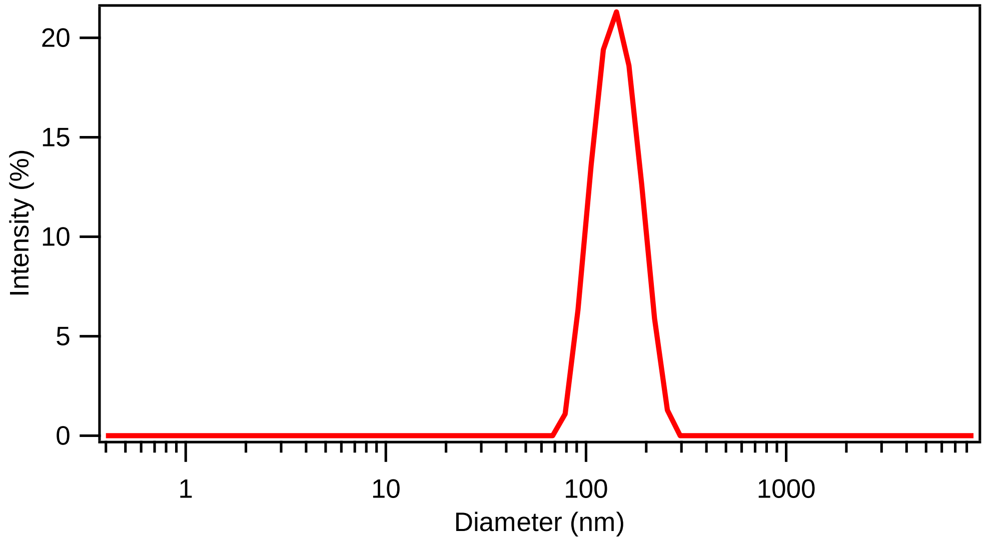

Figure S2: Size distribution measured by DLS for the DOPG liposome sample.

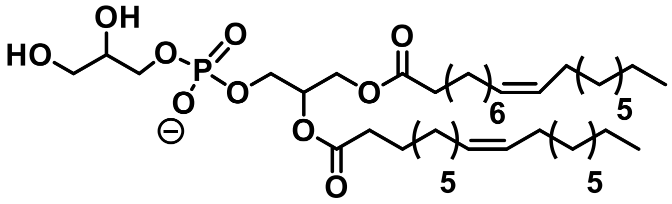

Figure S3. Molecular structure of DOPG.

Additional Details on SHG Measurements of Molecular Interactions with Liposomes

Representative second harmonic generation (SHG) spectra of the liposome and malachite green (MG) samples are shown in Figure S4a. The SHG spectrum of the DOPG liposomes upon addition of 15 μM MG at 25 °C at 50 μM lipid concentration in 5.0 mM citrate buffer of pH 4.0 shows a strong SHG signal centered at 400 nm with a full width at half maximum of 4.5 nm. The small signal at wavelengths greater than 410 nm is due to two-photon fluorescence from MG,[2,4] and is clearly separated spectroscopically from the SHG signal. Here, the SHG intensity is enhanced by approximately 2.5 times in comparison to the hyper-Raleigh scattering (HRS) signal generated from free dye molecules alone,[5,6] confirming molecular adsorption of MG to the liposome surface. In contrast, the SHG signal from the liposomes alone is much lower, in agreement with our previous studies.[2,3] For a direct comparison, all SHG intensities are normalized with respect to the DOPG liposomes upon addition of 15 μM MG at 25 °C. Figure S4b displays the SHG spectra of DOPG liposomes immediately after the addition of 15 μM MG under different temperatures ranging from 25 °C to 40 °C. The SHG intensity is found to decrease as temperature is increased, which is primarily attributed to the change in the orientational distribution of the dipole moment of the adsorbed MG dye molecules at the liposome interface,[7] as discussed in greater detail in the paper and later in the Supporting Information using results obtained from molecular dynamics (MD) simulations.

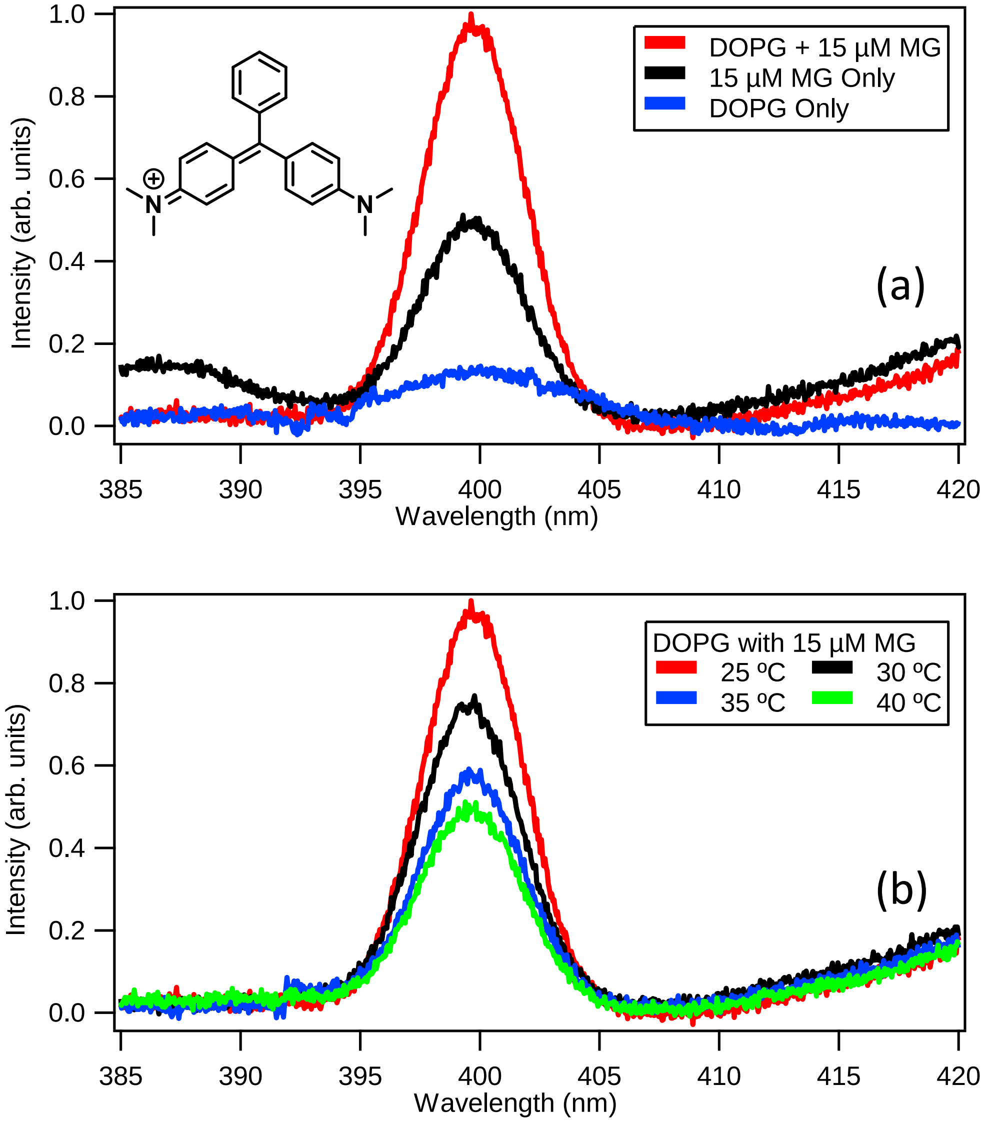

Figure S4. (a) SHG spectra of DOPG liposomes in the presence of 0 μM and 15 μM MG compared to 15 μM MG alone. Inset shows MG molecular structure. (b) SHG spectra of DOPG liposomes immediately upon the addition of 15 μM MG at different temperatures. All samples are in 5.0 mM citrate buffer at pH 4.0 with a lipid concentration of 50 μM.

The determined rate constants (τ^{-1}) for different temperatures are plotted as a function of MG concentration at different temperatures, as shown in Figure S5, with corresponding linear fits for each temperature. The slopes of obtained rate constants from Figure S5 are plotted as a function

of temperature and are displayed in Figure S6. SHG from a colloidal nanoparticle sample is understood to be a coherent process from each individual nanoparticle, while the overall SHG signal is the incoherent summation of an ensemble of nanoparticles at the laser focus, along with background signals from hyper Raleigh scattering.[5,8,9] Fitting the time traces using $E_{SHG}\ (t) = a_0 + a_1 e^{-t/\tau}$, as described in the paper, properly accounts for these coherent and incoherent signals. The transport times τ are tabulated in Table S1. The fitting parameters a_0 and a_1 are listed in Tables S2 and S3, respectively. The HRS intensities obtained for different MG concentrations in 5 mM citrate buffer with pH 4.0 at 25 °C and 40 °C are shown in Figure S7 with corresponding linear fits. The HRS signal is from incoherent second-order scattering which arises from orientational and density fluctuations of molecules in the bulk solutions.[5] The HRS signal has the

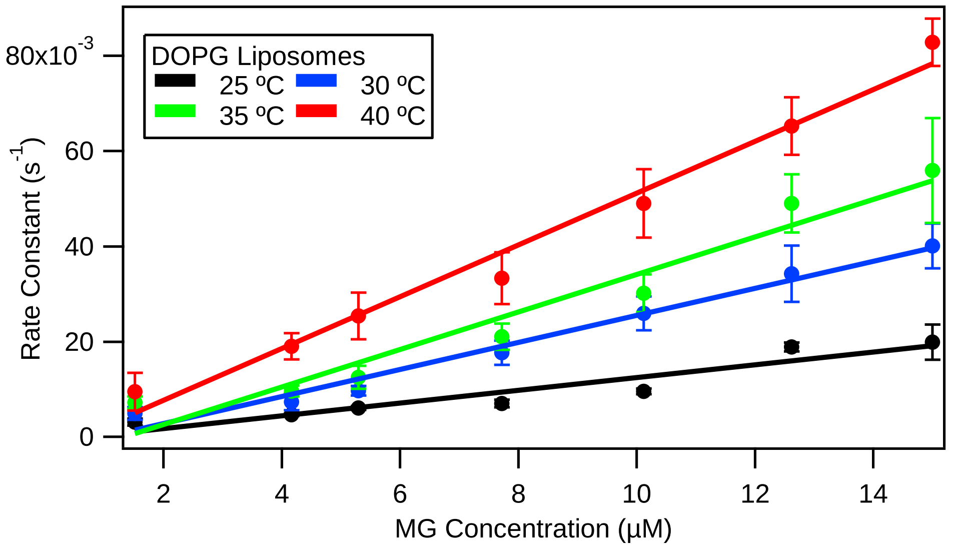

Figure S5. Variation of the transport rate constant as a function of MG concentration for DOPG liposomes with a lipid concentration of 50 μM in 5.0 mM citrate buffer of pH 4.0 at different temperatures with corresponding linear fits.

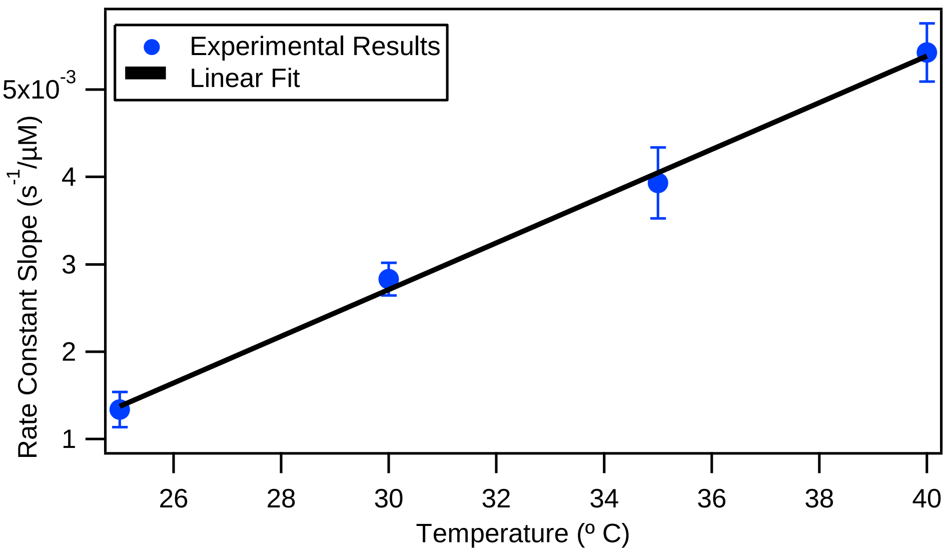

Figure S6. Variation of the rate constant slope as a function of temperature (blue circles) with a linear fit (black line) for DOPG liposomes with a lipid concentration of 50 μM in 5.0 mM citrate buffer of pH 4.0.

Table S1: Transport times of MG in DOPG liposomes at different temperatures

	Transport Time (s)			
**MG (μM)**	**25 °C**	**30 °C**	**35 °C**	**40 °C**
1.51	311 ± 72	199 ± 54	137 ± 23	104 ± 43
4.16	213 ± 11	134 ± 31	103 ± 12	52 ± 7
5.30	163 ± 11	102 ± 10	79 ± 15	39 ± 7
7.72	141 ± 15	56 ± 8	47 ± 6	29 ± 4
10.12	104 ± 6	38 ± 5	33 ± 4	20 ± 2
12.62	52 ± 2	29 ± 5	20 ± 2	15 ± 1
15.00	50 ± 9	24 ± 3	17 ± 3	12 ± 1

Table S2: Fitting Parameter a_0 of MG in DOPG liposomes at different temperatures

**Fitting Parameter a_0**				
**MG (μM)**	**25 °C**	**30 °C**	**35 °C**	**40 °C**
1.51	0.071 ± 0.007	0.072 ± 0.001	0.071 ± 0.002	0.072 ± 0.001
4.16	0.155 ± 0.002	0.161 ± 0.002	0.121 ± 0.001	0.109 ± 0.001
5.30	0.221 ± 0.004	0.259 ± 0.003	0.191 ± 0.001	0.134 ± 0.001
7.72	0.307 ± 0.002	0.316 ± 0.001	0.212 ± 0.002	0.122 ± 0.002
10.12	0.412 ± 0.001	0.377 ± 0.001	0.308 ± 0.001	0.231 ± 0.001
12.62	0.551 ± 0.002	0.462 ± 0.002	0.349 ± 0.001	0.263 ± 0.001
15.00	0.466 ± 0.001	0.505 ± 0.001	0.388 ± 0.001	0.323 ± 0.001

Table S3: Fitting Parameter a_1 of MG in DOPG liposomes at different temperatures

**Fitting Parameter a_1**				
**MG (μM)**	**25 °C**	**30 °C**	**35 °C**	**40 °C**
1.51	0.073 ± 0.005	0.054 ± 0.004	0.024± 0.002	0.030 ± 0.003
4.16	0.259 ± 0.004	0.120 ± 0.008	0.120 ± 0.005	0.066 ± 0.004
5.30	0.386 ± 0.011	0.218 ± 0.010	0.160 ± 0.007	0.148 ± 0.011
7.72	0.426 ± 0.011	0.239 ± 0.007	0.241 ± 0.009	0.237 ± 0.013
10.12	0.452 ± 0.009	0.275 ± 0.011	0.229 ± 0.006	0.195 ± 0.012
12.62	0.465 ± 0.018	0.296 ± 0.016	0.263 ± 0.006	0.220 ± 0.008
15.00	0.546 ± 0.009	0.318 ± 0.012	0.267 ± 0.007	0.235 ± 0.006

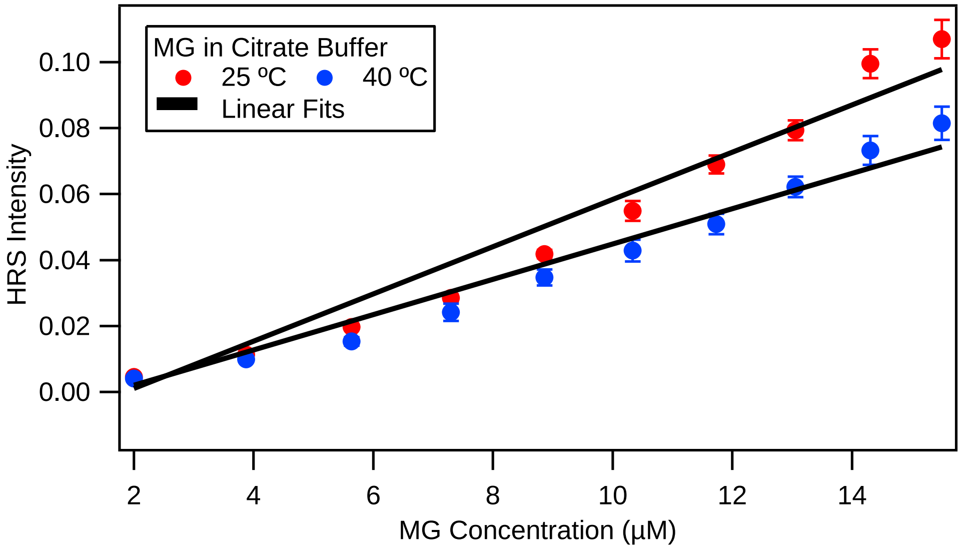

Figure S7. HRS signal intensity of 5.0 mM citrate buffer of pH 4.0 as a function of added MG concentration (blue and red circles) compared to linear fits (black lines) at 25 °C and 40 °C, respectively.

same spectra as the SHG signals, centered at 400 nm, and the same data acquisition and analysis procedures are used.

Second harmonic generation studies of colloidal polystyrene sulfate particles

Adsorption isotherms for MG adsorbing to the surface of polystyrene sulfate (PSS) microspheres in aqueous colloidal suspension are determined using SHG measurements for comparison to the liposome results presented in the paper. These results demonstrate the general applicability of temperature-dependent SHG adsorption isotherm measurements for determining enthalpy and entropy, while also providing relevant comparisons to the results from the DOPG liposomes. The PSS microspheres of diameter 1.06 ± 0.03 μm are purchased from Polysciences and are diluted in nanopure water to a concentration of 1.8×10^{10} particles/mL. The experimental setup is the same as the one used for the liposome measurements, with the same data analysis procedure. The adsorption of MG to polystyrene sulfate particles (PSSPs) in water is driven by the electrostatic

force between the positively-charged amine group of MG and the negatively-charged sulfate group at the PSSP surface.[9,10] The SHG adsorption isotherms for this system are studied as a function of temperature, with the results shown in Figure S8 for temperatures of 20 °C, 30 °C, 40 °C, 50 °C, and 60 °C. The experimentally obtained isotherms are fit using the modified Langmuir model, as described previously. The fitting parameters obtained from the modified Langmuir fits are listed in Table S2. The obtained equilibrium constant is a measure of the electrostatic interaction between the charged dye and microparticle interface. However, the value of the equilibrium constant does not change to within the experimental uncertainty for the temperature range studied here. The obtained adsorption equilibrium constants for 20 °C, 30 °C, 40 °C, 50 °C, and 60 °C are (2.37 ± 0.32) × 10^9, (1.9 ± 0.53) × 10^9, (2.49 ± 0.32) × 10^9, (2.81 ± 0.32) × 10^9, and (2.27 ± 0.54) × 10^9, respectively. In comparison to the liposome results presented in the manuscript, the obtained adsorption equilibrium constants are larger in magnitude indicating a stronger interaction between MG and PSSPs, in agreement with the previous studies.[3,11]

The free energy of adsorption, obtained from $\Delta G = -RT \ln K$, is plotted as a function of temperature, as shown in Figure S9. Here, the results are fit to a line with $\Delta G = \Delta H - T\Delta S$ to provide the thermodynamic properties of the molecular adsorption, where ΔH is the change in adsorption enthalpy, ΔS is the change in adsorption entropy, and T is the temperature. The calculated ΔH from the y-intercept is 0.45 ± 1.00 kcal/mol, indicating that the net change in adsorption enthalpy is approximately zero to within experimental uncertainty. The calculated ΔS from the linear slope is 0.044 ± 0.003 kcal/K·mol. This change in entropy is a full accounting of the adsorption process, including the change in entropy of the adsorbate molecules and the solvated microparticle surface. The adsorption process is described by free dye molecules adsorbing to "empty" adsorption sites, which are then converted to "filled" adsorption sites.[2,4,8] The molecular

adsorption of the MG adsorbates alone should have negative entropy as these MG molecules are more ordered when adsorbed to the nanoparticle surface. However, the adsorbate MG molecules are replacing water molecules and counterions that were originally at the nanoparticle surface. Since each MG adsorbate molecule will replace numerous water molecules and counterions due to their relative sizes, an overall increase of entropy occurs upon adsorption, when given a full account. For this particular case, where ΔH is approximately equal to zero to within the experimental uncertainty, the overall condition is $\Delta G \approx -T\Delta S$, so the molecular adsorption process is expected to be spontaneous at all aqueous temperatures.

The obtained values of N_{max} from the temperature-dependent SHG adsorption isotherms are 0.027 ± 0.001 μM, 0.070 ± 0.001 μM, 0.138 ± 0.016 μM, 0.162 ± 0.002 μM, and 0.237 ± 0.028 μM for 20 °C, 30 °C 40 °C, 50 °C, and 60 °C, respectively. The N_{max} values all increase with increasing temperature. This trend is in agreement with the liposomes results as well. It is important to point out that in comparison to PSSPs, liposomes systems are more complicated as both adsorption and transport takes place. Additionally, the N_{max} values increase approximately 10-fold as the temperature is increased from 20 °C to 60 °C indicating that the PSSP double layer is more susceptible to temperature changes than the lipid bilayer. As temperature increases, the collision frequency of MG and counterions at the surface increase. This may result in increased ion-pairing and decreased adsorbate-adsorbate repulsion as the temperature increases, leading to larger N_{max} values. The SHG intensities at the plateau region also increase as the temperature increases due to these larger N_{max} values. This trend is the opposite as compared to results obtained with the liposomes, illustrating the importance of considering both N_{max} and orientation angle at the colloidal surface.

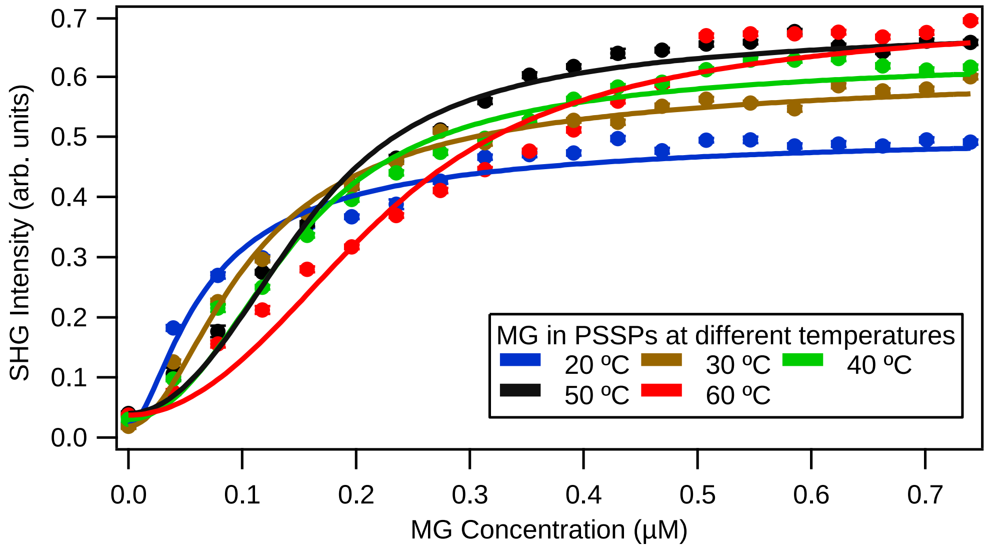

Figure S8. SHG-determined adsorption isotherms for MG with 1 μm polystyrene sulfate particles (PSSPs) in water at different temperatures.

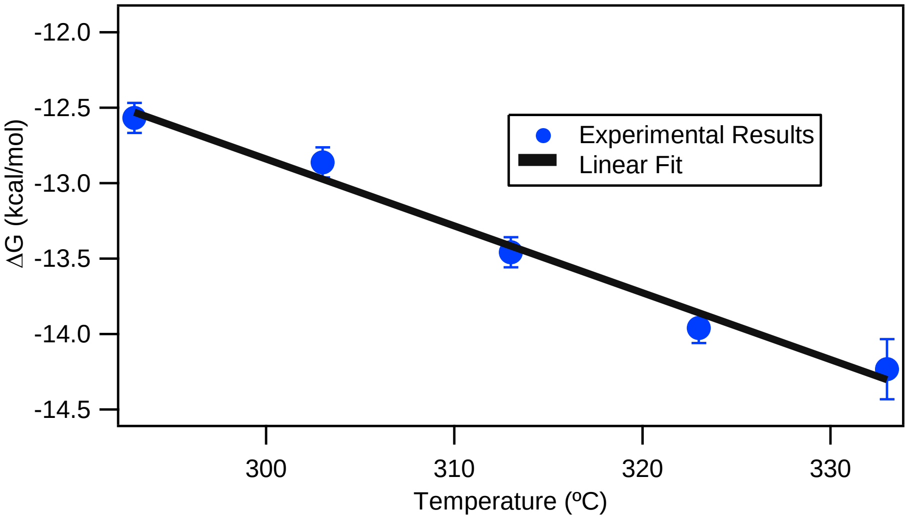

Figure S9. Adsorption free energy for MG to the PSSPs surface in water under varying temperature.

Table S4. List of Variables and Fitting Parameters Obtained from the Modified Langmuir Isotherm Model for MG to PSSPs at Different Temperatures

Temperature		K (10^9)	N_{max} (μM)	-ΔG (kcal/mol)
293 K	20°C	2.37 ± 0.32	0.027 ± 0.001	12.6 ± 0.1
303 K	30°C	1.90 ± 0.53	0.070 ± 0.001	12.8 ± 0.1
313 K	40°C	2.49 ± 0.37	0.138 ± 0.016	13.4 ± 0.1
323 K	50°C	2.81 ± 0.33	0.162 ± 0.002	13.9 ± 0.1
333 K	60°C	2.27 ± 0.54	0.237 ± 0.028	14.2 ± 0.2

Molecular Dynamics Simulations

The initial lipid bilayer with 72 DOPG molecules is built using the CHARMM-GUI Membrane Builder[12,13] followed by the solvation of the system with 6075 SPC/E[14] water molecules and 72 K^+ ions to neutralize the system charge using the Packmol[15] software package. The partial charges of the optimized structures are calculated by the RESP fitting technique[16,17] using the HF/6-31G* method in Gaussian 09 suite of programs.[18] The initial box dimensions of the system are 57.0 Å × 60.0 Å × 124.0 Å. The Moltemplate[19] package is used to generate the input files for the molecular dynamics simulations. Using the LAMMPS program (version 05 Sep 2014)[20] molecular dynamics simulations are carried out with the all-atom general AMBER force field (GAFF)[21] at a temperature of 303 K. In order to equilibrate the system, an initial energy minimization followed by a 5 ns simulation in the isothermal-isobaric (NPT) ensemble and then a 10 ns simulation in the canonical (NVT) ensemble are performed. The system is then replicated along the x-axis. This elongated system is further simulated for 5 ns in the canonical (NVT) ensemble making the final simulation box dimension approximately 94.5 Å × 50.0 Å × 112.0 Å.

The next steps of the simulation process are to introduce the MG molecule to the pre-equilibrated DOPG system followed by equilibration of the system at the two different temperatures. For this purpose the MG molecule is built using the Avogardo[22] package and the structure optimization and the partial charge calculations (by the RESP fitting technique[16,17]) are performed using the Gaussian 09[18] suite of programs with the HF/6-31G* method. The MG molecule is then added to a vacuum layer of length 15 Å in the z-direction of the pre-equilibrated DOPG membrane system. This system is then simulated at two different temperatures (303 K and 313 K). For each system, equilibration simulations in the isothermal-isobaric (NPT) ensemble for 5 ns and in the canonical (NVT) ensemble for 10 ns are performed. The final simulation box dimensions are approximately 94.5 Å × 50.0 Å × 115.0 Å for the system simulated at 303 K and 94.0 Å × 50.0 Å × 117.5 Å for the system simulated at 313 K. A simulation for another total of 10 ns follows these simulations. To maintain the temperatures at 303 K and 313 K, the Langevin thermostat[23] with a collision frequency of 1 ps^{-1} is applied. The pressure of the system is controlled semi-isotropically (only along the z-component of the system) which has been used previously by by Mario Orsi et al.[24] at 1 atm, using the Berendsen barostat[25] with a damping time of 1 ps and an isothermal compressibility of 4.6×10^{-5} atm^{-1} in both simulations. Periodic boundary conditions with the SHAKE algorithm are used for these simulations for the water molecules to restrain the structure of the water molecules. Electrostatic interactions are calculated using the particle-particle particle-mesh (PPPM)[26] method and the non-bonded Lennard-Jones interactions are cutoff beyond 10 Å. A 1 fs time step is used for both the simulations at the two temperatures.

In order to obtain the free energy profile of the adsorption process of the MG molecule onto the DOPG membrane at the two different temperatures, an enhanced sampling technique, namely the umbrella sampling method,[27] is used. The final structures from the canonical

simulations performed before are used as the starting configurations for the umbrella sampling windows. For these umbrella sampling simulations, the distance in the z-direction between the center of mass (COM) of the MG molecule and the COM of the DOPG membrane is used as the collective variable. For each system, 32 umbrella sampling windows (each with 18 ns) are simulated, with a step-size of 1.5 Å and a harmonic force constant of 3 kcal/mol. To derive the potential of mean force (free energy) from the umbrella sampling simulations, the weighted histogram analysis (WHAM)[28,29] method is used. For this process, only the last 14 ns of each umbrella sampling window is used. Using the block averaging method with 3.5 ns of simulation time for each block, the statistical error with the potential of mean force is calculated. The average surface of the DOPG membrane is determined using the average z-coordinates of the oxygen atom of each DOPG's furthest hydroxyl group on one side of the membrane. In addition, the change in the normalized water number density is used to determine the membrane-water interface. For this analysis the umbrella sampling window when the COM of the MG molecule is approximately 25 Å away from the average membrane surface is used. In order to obtain the water number density, the number of water molecules in a water layer with 1 Å thickness in the z-direction is counted and binned.

Canonical Simulations

Prior to the umbrella sampling simulations, 20 ns of canonical simulations are performed. Figure S10 shows the distance in the z-direction between the COM of the MG molecule and the COM of the DOPG membrane for the canonical simulations for the two different temperatures. The average surface of the DOPG membrane is determined using the average z-coordinates of the oxygen atom of each DOPG's furthest hydroxyl group on one side of the membrane and the interfacial region is determined using the normalized water number density.

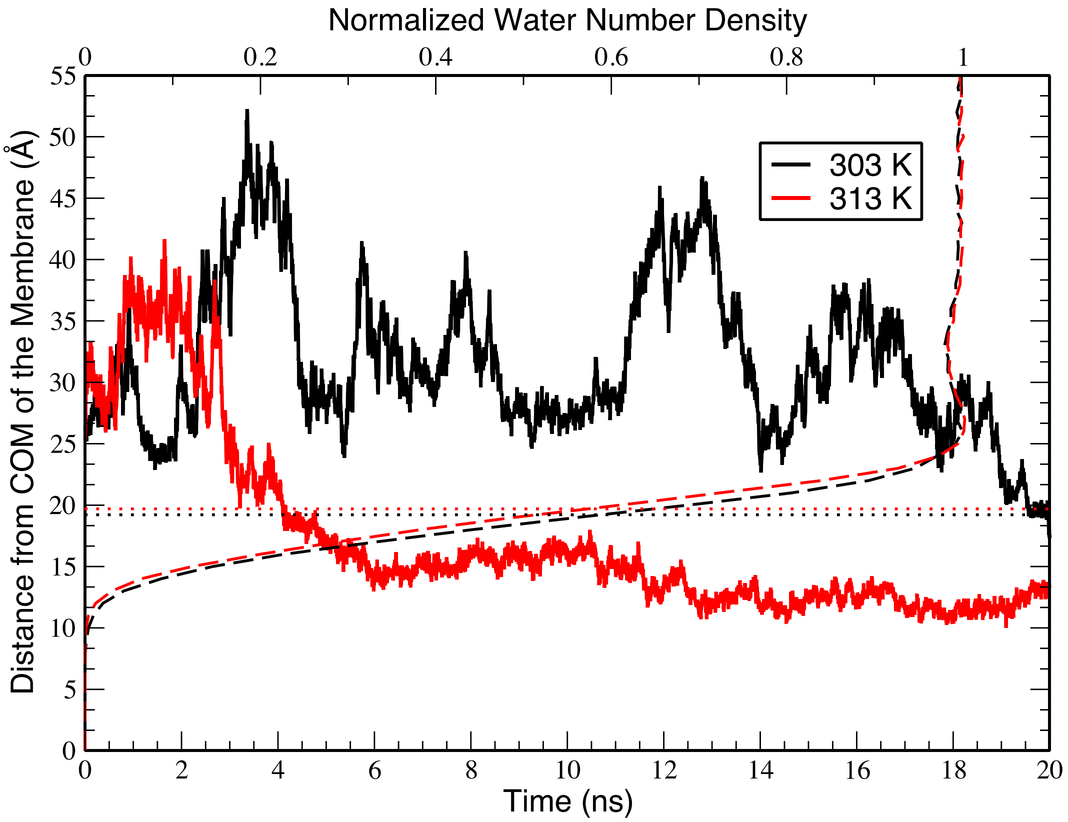

Figure S10. The distance in the z-direction between the COM of the MG molecule and the COM of the DOPG membrane for 303 K and 313 K temperature for the canonical simulations. The horizontal dotted lines represent the average interface for 303 K (black) and 313 K (red). The dashed lines represent the normalized water number density for 303 K (black) and 313 K (red).

According to results in Figure S10, the MG molecule gets adsorbed more rapidly to the DOPG membrane at the higher temperature compared to the lower temperature simulation. Figure S11 shows a representative snapshot of the MG molecule adsorbed on to the DOPG membrane during the canonical simulation at 313 K temperature. The MG molecule at the lower temperature takes much longer to reach the interface and does not readily penetrate the membrane, unlike in the higher temperature case. This indicates that the higher energy barrier at the lower temperature

simulation hinders the adsorption process of the MG molecule as compared to the higher temperature simulation.

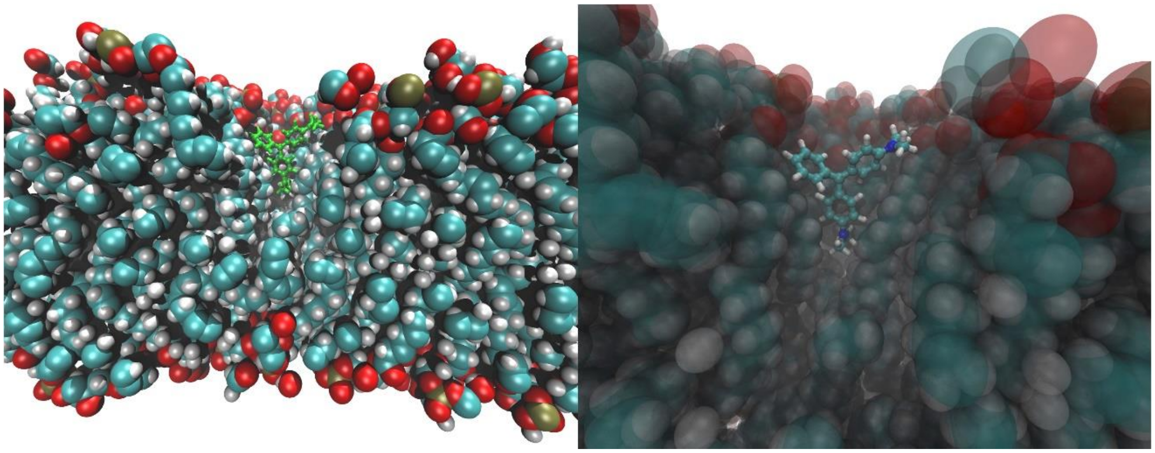

Figure S11. A representative snapshot of the MG molecule adsorbed on to the DOPG membrane during the canonical simulation under 313 K temperature (on the left) and a magnified view of the snapshot (on the right).

Orientation Calculations

To gain a molecular level understanding of the change of SHG intensities at different temperatures, the orientation angle θ (Figure S12a) between the dipole moment vector V_a of the MG molecule and the vector normal to the membrane surface V_s pointing to the solvent side, with the MG molecule at different distances in the z-direction from the COM of the membrane is calculated. The results from this analysis are shown in Figure S12. This specific type of orientational distribution has been used to gain an understanding of molecular ordering at interfaces in previous MD studies.[30] Figure S12b shows that the dipole vector of the MG molecule always forms a narrow distribution at an angle greater than 90°, with the two amine groups of the MG molecule directed towards the membrane, at the lower temperature. This demonstrates that the orientation of the MG molecule is well ordered at the aqueous solution-membrane interface at the lower temperature,

thereby enhancing the intensity of the SHG signal. In contrast, at the higher temperature, the angular distribution is much broader with a wider range of angles indicating a less-ordered interfacial structure due to the weakened interaction between the MG and the DOPG molecules, as shown in Figure S12c. This contributes to the lower observed SHG intensity at higher temperatures. It should be noted that adsorbate-adsorbate interactions and additional interactions with added salt and buffer molecules are present in the experimental studies, but are not included in the MD studies. The MD simulations are also used to study the displacement of the water solvent molecules caused by the MG molecular adsorption, as explained in greater detail in the section below. According to the results shown in Figure S13, approximately 50 – 70 water molecules are displaced when MG adsorbs to the DOPG membrane at both 303 K and 313 K. This is also in agreement with the SHG experimental results, where a positive change in entropy is observed due to adsorption from a large number of interfacial water molecules being displaced from every singular MG molecular adsorption event that occurs.

Water and Potassium Ion Displacement Calculations

To analyze the displacement of water molecules as the MG molecule adsorbs to the DOPG membrane, the change in the number of interfacial water molecules is calculated. This interfacial water layer is defined as the water molecules which are within 3.5 Å along the z-direction from the average surface of the membrane. The average surface of the DOPG membrane is determined using the z-coordinates of the oxygen atom of each DOPG's furthest hydroxyl group on one side of the membrane. Figure S13 shows the number of water molecules in the layer within 3.5 Å from the average surface of the membrane for different umbrella sampling windows calculated as a function of the separation in the z-direction between the MG molecule and COM of the membrane. According to these results, the number of water molecules decreases more rapidly at the higher

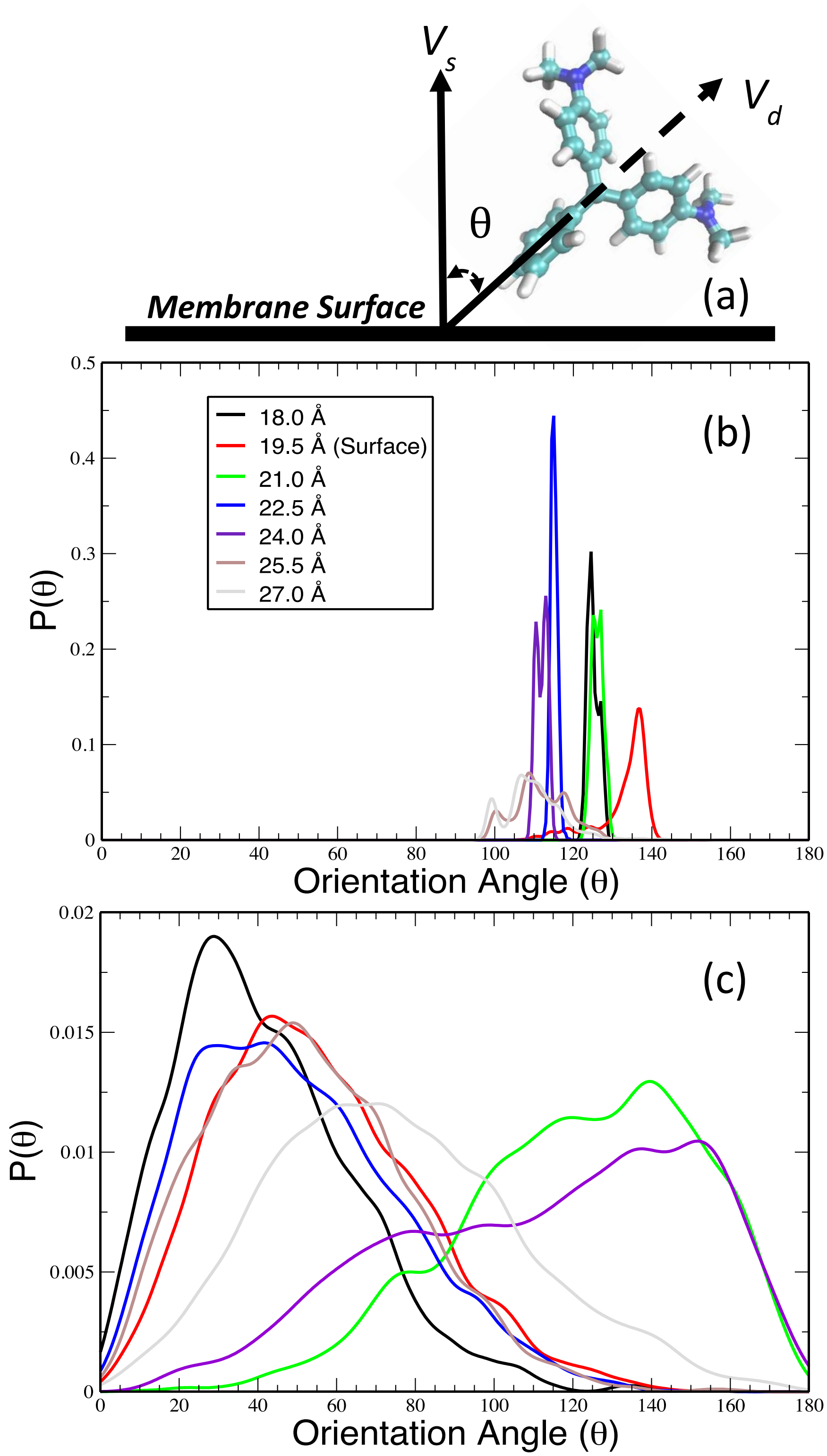

Figure S12. (a) Definition of the orientation angle θ of MG. V_d is the dipole vector of the MG molecule, and V_s is the vector normal to the DOPG membrane surface, pointing in the direction of the solvent side. The probability distribution of the angle θ for different umbrella sampling windows at (b) 303 K and (c) 313 K. The legend in (b) indicates the distance in the z-direction between the COM of the DOPG membrane and the COM of the MG molecule for both (b) and (c).

temperature as the MG molecule approaches the membrane, as compared to the lower temperature case. This is consistent with the orientation angle distributions observed for the dye at two temperatures. For the higher temperature the orientation angle distribution is much broader and more random while it is more ordered in the lower temperature simulations. Since the dipole vector distribution is broader at higher temperature, the MG molecule clearly shows greater orientational variability during adsorption to the membrane, which results in more water molecules being displaced near the membrane surface at the higher temperature.

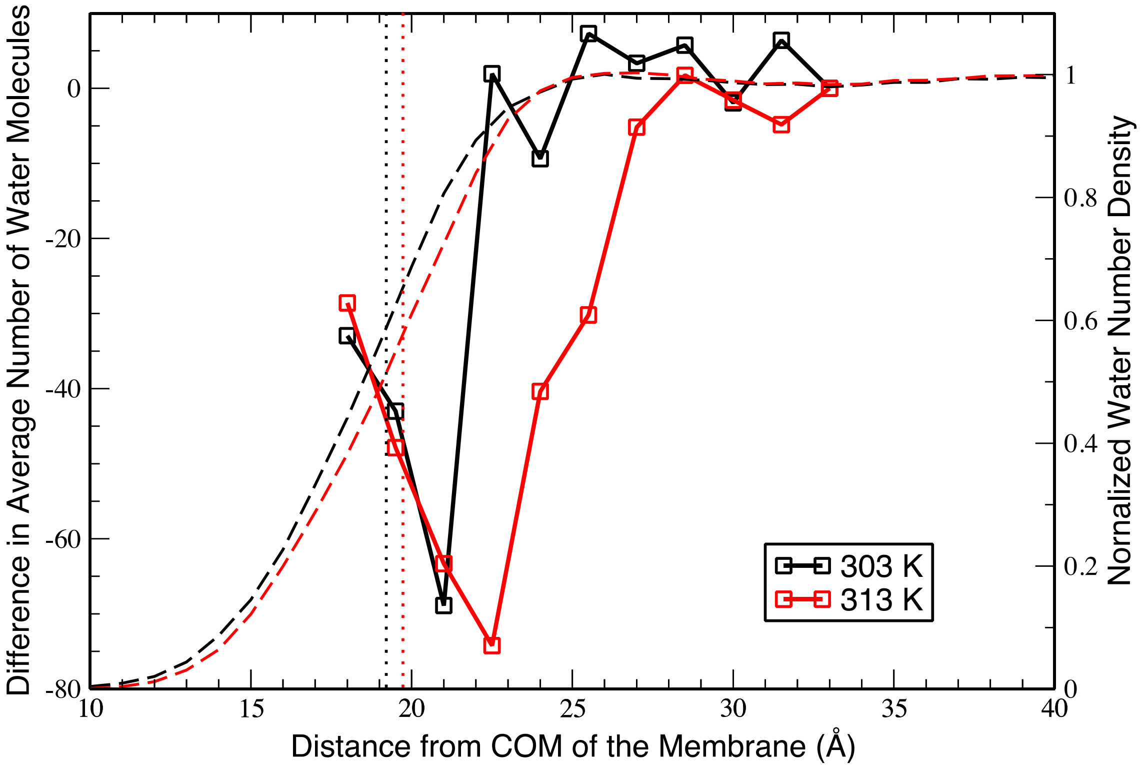

Figure S13. The number of water molecules in the first solvation layer of the membrane, when the MG molecule is at different separation distances in the z-direction from the COM of the DOPG membrane for temperatures of 303 K and 313 K. The vertical dotted lines represent the average interface for 303 K (black) and 313 K (red). The dashed lines represent the normalized water number density change for 303 K (black) and 313 K (red).

The number of water molecules displaced by MG adsorption is also consistent with a computational study of monatomic ion adsorption at the air-water interface.[31] This previous study calculated the average number of surface water molecules as a monoatomic cation of the size of a potassium ion approaches the air-water interface, finding that approximately 6 water molecules are displaced for each cation that is adsorbed. The volume of the monoatomic cation, using the K^+ van der Waals radius, can be compared to the volume of the MG molecular cation that is adsorbed in our study. The volume of the MG molecule can be crudely approximated by $V = \sum_{i=1}^{i=N} 4\pi r_i^3 . n_N$, where V is the total atomic volume, r_i is the van der Waals radius of atom i, and n is the number of atoms in each atom type. Using the van der Waals radius in Å, with values 1.7 Å for the C atom, 1.2 Å for the H atom, and 1.55 Å for the N atom, gives an overall volume of 162.95 Å^3 for the MG molecule. This compares to a volume of 13.25 Å^3 for the K^+ cation, with a van der Waals radius of 2.27 Å. The MG molecule is approximately 12 times larger than the potassium ion, so approximately 72 water molecules should get displaced with the adsorption of the MG molecule, based on this estimate, which is in general agreement with our MD results.

For comparison, the displacement of K^+ ions is also calculated as the MG molecule approaches the DOPG membrane. The number of interfacial K^+ ions is calculated as a function of distance in the z-direction of the MG molecule from the COM of the membrane. This interfacial K^+ ion layer is defined as the K^+ ions which are within 3.5 Å along the z-direction from the average surface of the membrane. Figure S14 shows the number of K^+ ions in the layer within 3.5 Å from the average surface of the membrane for different umbrella sampling windows calculated as a function of the separation in the z-direction between the MG molecule and COM of the membrane. According to these results, the change in the number of K^+ ions as the MG molecule approaches the membrane is minimal, fluctuating between an average number of 33 and 40, with a net

difference of approximately 1 or 2 (changing from approximately 40 to 39 at 313 K, and changing from approximately 39 to 37 at 303 K). This result shows that the number of K^+ ions displaced (~1) is much less than the number of water molecules displaced (~70) as the MG molecule adsorbs to the DOPG membrane.

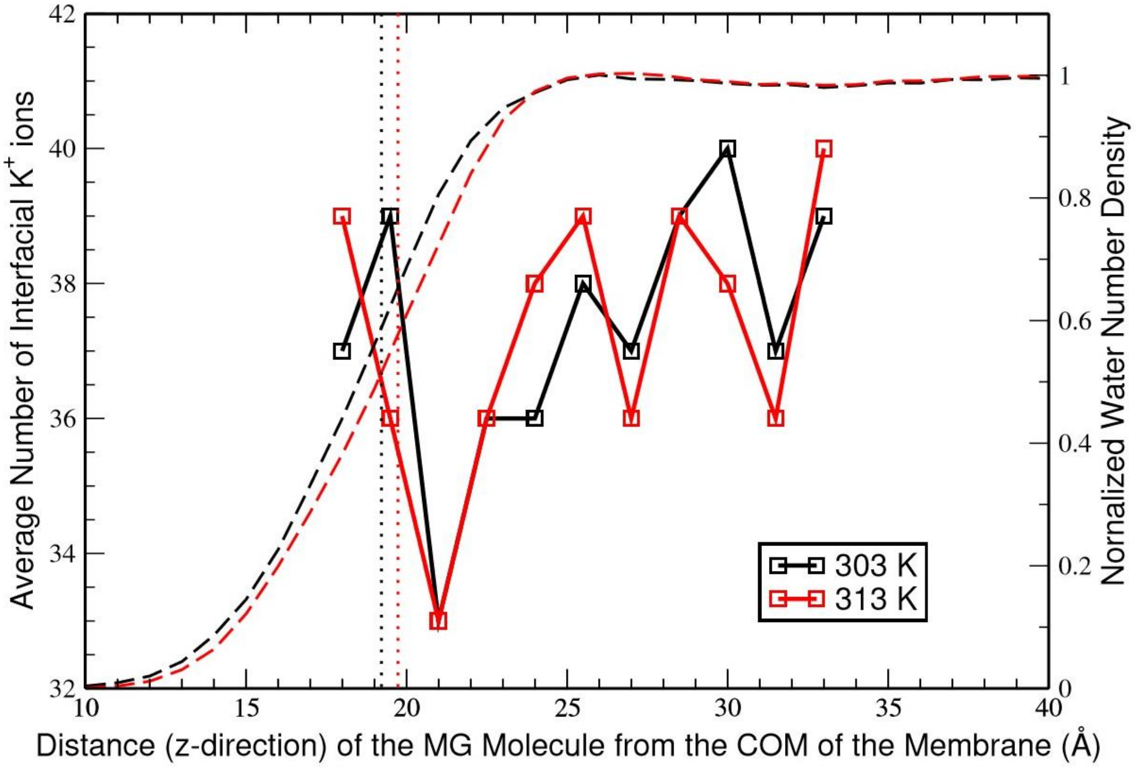

Figure S14. The number of K^+ in the first solvation layer of the membrane, when the MG molecule is at different separation distances in the z-direction from the COM of the DOPG membrane for temperatures of 303 K and 313 K. The vertical dotted lines represent the average interface for 303 K (black) and 313 K (red). The dashed lines represent the normalized water number density change for 303 K (black) and 313 K (red).

Error Analysis of Fits

The R^2-values obtained for time-dependent exponential fits of the SHG results from Figure 2 are summarized in Table S4. Similarly, the R^2-values obtained for the modified Langmuir fits of the SHG results for the liposomes and PSSPs are shown in Tables S6 and S7, respectively.

Table S5: R^2 values of time-dependent exponential fits of the SHG results

R^2-values of exponential fits				
**MG (μM)**	**25 °C**	**30 °C**	**35 °C**	**40 °C**
1.51	0.999	0.994	0.997	0.997
4.16	0.998	0.996	0.998	0.998
5.30	0.996	0.997	0.999	0.995
7.72	0.998	0.999	0.998	0.991
10.12	0.999	0.999	0.999	0.997
12.62	0.999	0.999	0.999	0.999
15.00	0.999	0.999	0.999	0.999

Table S6: R^2 values of modified Langmuir fits from SHG results for liposomes

**Temperature**	**25 °C**	**30 °C**	**35 °C**	**40 °C**
R^2 –value	0.989	0.993	0.995	0.989

Table S7: R^2 values of modified Langmuir fits from SHG results for PSSPs

Temperature	20 °C	30 °C	40 °C	50 °C	60 °C
R^2 –value	0.964	0.989	0.978	0.988	0.966

References

(1) Ong, W.; Yang, Y.; Cruciano, A. C.; McCarley, R. L. Redox-triggered contents release from liposomes. *J. Am. Chem. Soc.* **2008**, *130*, 14739-14744.

(2) Kumal, R. R.; Nguyenhuu, H.; Winter, J. E.; McCarley, R. L.; Haber, L. H. Impacts of Salt, Buffer, and Lipid Nature on Molecular Adsorption and Transport in Liposomes As Observed by Second Harmonic Generation. *Journal of Physical Chemistry C* **2017**, *121*, 15851-15860.

(3) Hamal, P.; Nguyenhuu, H.; Subasinghege Don, V.; Kumal, R. R.; Kumar, R.; McCarley, R. L.; Haber, L. H. Molecular Adsorption and Transport at Liposome Surfaces Studied by Molecular Dynamics Simulations and Second Harmonic Generation Spectroscopy. *J. Phys. Chem. B* **2019**, *123*, 7722-7730.

(4) Karam, T. E.; Haber, L. H. Molecular adsorption and resonance coupling at the colloidal gold nanoparticle interface. *Journal of Physical Chemistry C* **2014**, *118*, 642-649.

(5) Roke, S.; Gonella, G. Nonlinear light scattering and spectroscopy of particles and droplets in liquids. *Annu. Rev. Phys. Chem.* **2012**, *63*, 353-378.

(6) Rao, Y.; Guo, X.-m.; Tao, Y.-S.; Wang, H.-f. Observation of the direct S 2→ S 0 two-photon fluorescence between 370 and 480 nm and the hyperpolarizability of crystal violet (CV) from spectrally resolved hyper-Rayleigh scattering measurement. *J. Phys. Chem. A* **2004**, *108*, 7977-7982.

(7) Tran, R. J.; Sly, K. L.; Conboy, J. C. Applications of surface second harmonic generation in biological sensing. *Annual Review of Analytical Chemistry* **2017**, *10*, 387-414.

(8) Eisenthal, K. B. Second harmonic spectroscopy of aqueous nano-and microparticle interfaces. *Chem. Rev.* **2006**, *106*, 1462-1477.

(9) Gonella, G.; Dai, H.-L. Second harmonic light scattering from the surface of colloidal objects: theory and applications. *Langmuir* **2013**, *30*, 2588-2599.

(10) Wang, H.; Yan, E. C.; Liu, Y.; Eisenthal, K. B. Energetics and population of molecules at microscopic liquid and solid surfaces. *J. Phys. Chem. B* **1998**, *102*, 4446-4450.

(11) Zeng, J.; Eckenrode, H. M.; Dai, H.-L.; Wilhelm, M. J. Adsorption and transport of charged vs. neutral hydrophobic molecules at the membrane of murine erythroleukemia (MEL) cells. *Colloids Surf., B* **2015**, *127*, 122-129.

(12) Jo, S.; Kim, T.; Iyer, V. G.; Im, W. CHARMM-GUI: a web-based graphical user interface for CHARMM. *J. Comput. Chem.* **2008**, *29*, 1859-1865.

(13) Wu, E. L.; Cheng, X.; Jo, S.; Rui, H.; Song, K. C.; Dávila-Contreras, E. M.; Qi, Y.; Lee, J.; Monje-Galvan, V.; Venable, R. M. CHARMM-GUI membrane builder toward realistic biological membrane simulations. *J. Comput. Chem.* **2014**, *35*, 1997-2004.

(14) Berendsen, H.; Grigera, J.; Straatsma, T. The missing term in effective pair potentials. *J. Phys. Chem.* **1987**, *91*, 6269-6271.

(15) Martínez, L.; Andrade, R.; Birgin, E. G.; Martínez, J. M. PACKMOL: a package for building initial configurations for molecular dynamics simulations. *J. Comput. Chem.* **2009**, *30*, 2157-2164.

(16) Bayly, C. I.; Cieplak, P.; Cornell, W.; Kollman, P. A. A well-behaved electrostatic potential based method using charge restraints for deriving atomic charges: the RESP model. *J. Phys. Chem.* **1993**, *97*, 10269-10280.

(17) Cieplak, P.; Cornell, W. D.; Bayly, C.; Kollman, P. A. Application of the multimolecule and multiconformational RESP methodology to biopolymers: Charge derivation for DNA, RNA, and proteins. *J. Comput. Chem.* **1995**, *16*, 1357-1377.

(18) M. J. Frisch, G. W. T., H. B. Schlegel, G. E. Scuseria, M. A. Robb, J. R. Cheeseman, G. Scalmani, V. Barone, B. Mennucci, G. A. Petersson, et. al. *Gaussian 09, Revision A.02* **2009**.

(19) Jewett, A. I.; Zhuang, Z.; Shea, J.-E. Moltemplate a coarse-grained model assembly tool. *Biophys. J.* **2013**, *104*, 169a.

(20) Plimpton, S. Fast parallel algorithms for short-range molecular dynamics. *Journal of computational physics* **1995**, *117*, 1-19.

(21) Wang, J.; Wolf, R. M.; Caldwell, J. W.; Kollman, P. A.; Case, D. A. Development and testing of a general amber force field. *J. Comput. Chem.* **2004**, *25*, 1157-1174.

(22) Hanwell, M. D.; Curtis, D. E.; Lonie, D. C.; Vandermeersch, T.; Zurek, E.; Hutchison, G. R. Avogadro: an advanced semantic chemical editor, visualization, and analysis platform. *Journal of cheminformatics* **2012**, *4*, 17.

(23) Schneider, T.; Stoll, E. Molecular-dynamics study of a three-dimensional one-component model for distortive phase transitions. *Physical Review B* **1978**, *17*, 1302.

(24) Ding, W.; Palaiokostas, M.; Wang, W.; Orsi, M. Effects of lipid composition on bilayer membranes quantified by all-atom molecular dynamics. *Journal of Physical Chemistry B* **2015**, *119*, 15263-15274.

(25) Berendsen, H. J.; Postma, J. v.; van Gunsteren, W. F.; DiNola, A.; Haak, J. R. Molecular dynamics with coupling to an external bath. *The Journal of chemical physics* **1984**, *81*, 3684-3690.

(26) Hockney, R. W.; Eastwood, J. W.: *Computer simulation using particles*; crc Press, 1988.

(27) Kästner, J. Umbrella sampling. *Wiley Interdisciplinary Reviews: Computational Molecular Science* **2011**, *1*, 932-942.

(28) Souaille, M.; Roux, B. t. Extension to the weighted histogram analysis method: combining umbrella sampling with free energy calculations. *Comput. Phys. Commun.* **2001**, *135*, 40-57.

(29) Kumar, S.; Rosenberg, J. M.; Bouzida, D.; Swendsen, R. H.; Kollman, P. A. The weighted histogram analysis method for free-energy calculations on biomolecules. I. The method. *J. Comput. Chem.* **1992**, *13*, 1011-1021.

(30) Subasinghege Don, V.; David, R.; Du, P.; Milet, A.; Kumar, R. Interfacial Water at Graphene Oxide Surface: Ordered or Disordered? *J. Phys. Chem. B* **2019**, *123*, 1636-1649.

(31) Wang, Y.; Sinha, S.; Desai, P. R.; Jing, H.; Das, S. Ion at air–water interface enhances capillary wave fluctuations: Energetics of ion adsorption. *J. Am. Chem. Soc.* **2018**, *140*, 12853-12861.
